# Supplementary material for: Dissecting surveying behavior of reactive microglia under chronic neurodegeneration
Source: bioRxiv. 2025 Jun 6:2025.06.02.657382. Preprint. [Version 2] doi: 10.1101/2025.06.02.657382 (PMC12157537; doi:10.1101/2025.06.02.657382)
Supplement: Supplement 1 [file media-1.pdf]

Supplementary Materials

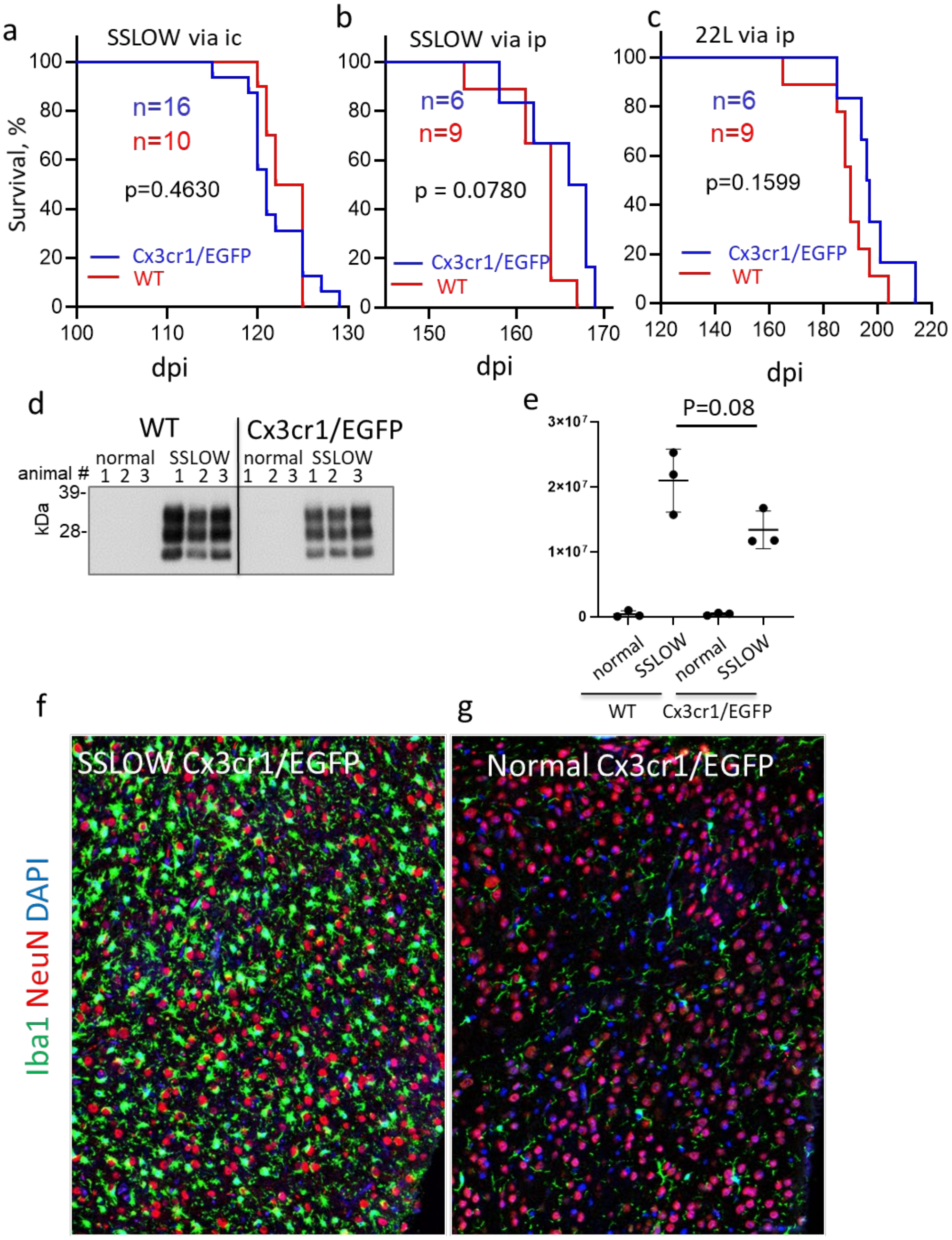

Figure S1. Prion pathogenesis in not changed in Cx3cr1/EGFP mice. a,b,c Survival curves for Cx3cr1/EGFP and WT (C57Bl/6J) mice inoculated with SSLOW via ic route (**a**), SSLOW via ip route (**b**) or 22L via ip route (**c**). Comparison by Mantel-Cox test. **d,e** Representative Western blot image (**d**) and quantification of PrP^{Sc} (**e**) in brains of WT and Cx3cr1/EGFP mice infected via ip route. The data presented as Means ± SD; p by Brown-Forsythe and Welch ANOVA with Dunnett's multiple comparison test, n=3 per group. Data for non-infected WT and Cx3cr1/EGFP brains (normal) are shown as a reference. **f, g** Immunostaining for microglia (Iba1, green) and neurons (NeuN, red) showing reactive Iba1+ cells enveloping neurons in cortex of Cx3cr1/EGFP mice infected by SSLOW via ip route at the terminal stage of the disease (**f**); and lack of neuronal envelopment in adult, non-infected Cx3cr1/EGFP mice (**g**).

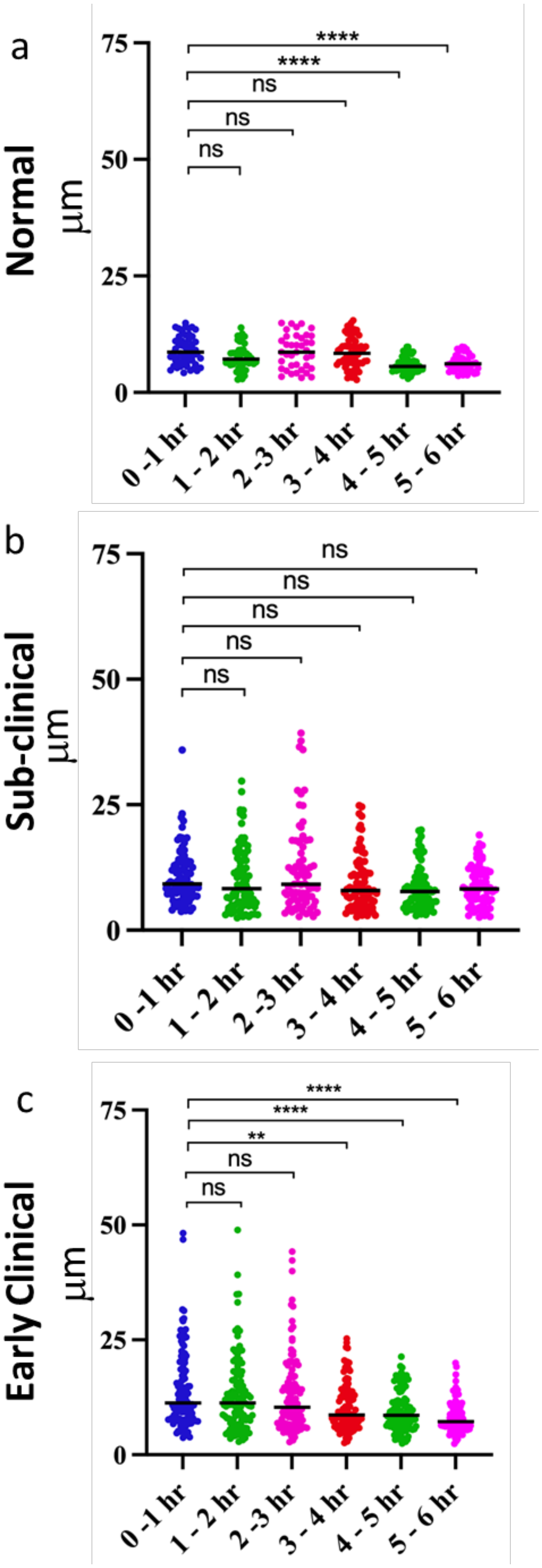

Figure S2. Mobility of EGFP$^+$ cells across six consecutive one-hour intervals post-slicing. Brain slices were prepared acutely using non-infected Cx3cr1/EGFP (normal) mice or Cx3cr1/EGFP mice infected with SSLOW via ip route at sub-clinical and early clinical stages of the disease. Distance covered by individual EGFP$^+$ cells in one-hour periods across six consecutive time intervals. Means are marked by black lines. n=40-65 cells per group, **$p<0.01$, ****$p<0.0001$, ns - non-significant by non-parametric Kruskal-Wallis test with Dunn's multiple comparison test.
